# Changes in the incidence of seasonal influenza in response to COVID-19 social distancing measures: an observational study based on Canada’s national influenza surveillance system

**DOI:** 10.17269/s41997-021-00509-4

**Published:** 2021-05-28

**Authors:** Andrew Pierce, Margaret Haworth-Brockman, Diana Marin, Zulma V. Rueda, Yoav Keynan

**Affiliations:** 1 National Collaborating Centre for Infectious Diseases, Rady Faculty of Health Sciences, University of Manitoba, Winnipeg, Manitoba Canada; 2 Department of Community Health Sciences, Max Rady College of Medicine, University of Manitoba, Winnipeg, Canada; 3 Facultad de Medicina, Universidad Pontificia Bolivariana, Medellín, Colombia; 4 Department of Medical Microbiology and Infectious Diseases, Max Rady College of Medicine, University of Manitoba, Winnipeg, Canada; 5 Section of Infectious Diseases, Department of Internal Medicine, Max Rady College of Medicine, University of Manitoba, Winnipeg, Canada

**Keywords:** Seasonal influenza, COVID-19, Coronavirus, Social distance, Canada, Grippe saisonnière, COVID-19, coronavirus, distance sociale, Canada

## Abstract

**Objectives:**

Seasonal influenza is an acute respiratory infection that presents a significant annual burden to Canadians and the Canadian healthcare system. Social distancing measures that were implemented to control the 2019–2020 novel coronavirus outbreak were investigated for their ability to lessen the incident cases of seasonal influenza.

**Methods:**

We conducted an ecological study using data from Canada’s national influenza surveillance system to investigate whether social distancing measures to control COVID-19 reduced the incident cases of seasonal influenza. Data taken from three separate time frames facilitated analysis of the 2019–2020 influenza season prior to, during, and following the implementation of COVID-19-related measures and enabled comparisons with the same time periods during three preceding flu seasons. The incidence, which referred to the number of laboratory-confirmed cases of specific influenza strains, was of primary focus. Further analysis determined the number of new laboratory-confirmed influenza or influenza-like illness outbreaks.

**Results:**

Our results indicate a premature end to the 2019–2020 influenza season, with significantly fewer cases and outbreaks being recorded following the enactment of many COVID-19 social distancing policies. The incidence of influenza strains A (H3N2), A (unsubtyped), and B were all significantly lower at the tail end of the 2019–2020 influenza season as compared with preceding seasons (*p* = 0.0003, *p* = 0.0007, *p* = 0.0019).

**Conclusion:**

Specific social distancing measures and behaviours may serve as effective tools to limit the spread of influenza transmission moving forward, as they become more familiar.

**Supplementary Information:**

The online version contains supplementary material available at 10.17269/s41997-021-00509-4.

## Résumé

**Objectifs:**

La grippe saisonnière est une infection aiguë des voies respiratoires qui représente un important fardeau annuel pour la population et pour le système de soins de santé du Canada. Nous avons cherché à déterminer si les mesures de distanciation sociale appliquées pour contrôler l’éclosion du nouveau coronavirus en 2019–2020 ont pu atténuer les cas incidents de grippe saisonnière.

**Méthodes:**

Nous avons mené une étude écologique à l’aide des données du système national de surveillance de l’activité grippale au Canada pour déterminer si les mesures de distanciation sociale appliquées pour contrôler la COVID-19 ont réduit les cas incidents de grippe saisonnière. Des données provenant de trois périodes distinctes ont facilité l’analyse de la saison grippale 2019–2020 avant, pendant et après l’application des mesures liées à la COVID-19 et permis des comparaisons avec les mêmes intervalles durant trois saisons grippales antérieures. L’incidence, c’est-à-dire le nombre de cas confirmés en laboratoire de souches grippales particulières, était notre principal objectif. Des analyses plus poussées ont permis de déterminer le nombre de nouvelles éclosions de syndrome grippal ou de grippe confirmée en laboratoire.

**Résultats:**

Selon nos résultats, la saison grippale 2019–2020 a connu une fin prématurée; un nombre sensiblement moins élevé de cas et d’éclosions ont été enregistrés après la mise en place des nombreuses politiques de distanciation sociale liées à la COVID-19. L’incidence des souches grippales A (H3N2), A (non sous-typée) et B a été sensiblement moins élevée vers la fin de la saison grippale 2019–2020 comparativement aux saisons précédentes (*p* = 0,0003, *p* = 0,0007, *p* = 0,0019).

**Conclusion:**

Des mesures et des comportements de distanciation sociale particuliers peuvent être des outils efficaces pour limiter la propagation de la grippe maintenant qu’ils sont plus familiers.

## Introduction

Seasonal influenza (flu) is an acute respiratory infection caused by the Orthomyxoviridae family of influenza viruses (Infection Prevention and Control Canada, 2020). It is often characterized by the sudden onset of cough, headache, fever, chills, myalgia, fatigue, and a general feeling of malaise (Infection Prevention and Control Canada, 2020). While forms of the illness can range from mild to severe, the influenza virus presents as a significant burden worldwide, accounting for up to 650,000 respiratory deaths annually (Iuliano et al., 2018). Influenza, along with pneumonia, consistently remains one of the top ten leading causes of death in Canada and is responsible for nearly 12,200 hospitalizations and 3500 deaths each year (Government of Canada, 2020c). These figures are likely underestimated, as laboratory confirmation varies according to age and clinical context, and it remains challenging to determine the extent to which influenza directly or indirectly contributes to health conditions that influence overall mortality (National Institute on Ageing, 2018).

Currently, the World Health Organization (WHO) recommends annual vaccination as the most effective way to prevent infection and severe outcomes caused by influenza viruses (World Health Organization, 2020a, b, c). This is especially true for those who may be particularly vulnerable to contracting severe forms of influenza, such as children under 5, people over 65, pregnant women, individuals living with chronic medical conditions, those who are otherwise immunocompromised, residents of long-term care facilities, and healthcare workers (Public Health Agency of Canada, 2016). Vaccination, however, remains moderately effective as it is highly dependent on the match between the circulating strain of virus and the vaccine, as well as the uptake and characteristics of those being vaccinated. For example, in Canada, annual vaccine coverage among adults varies: vaccination coverage was 42% in the 2018–2019 season, 38% in 2017–2018, and 36% in 2016–2017, with higher rates in women (women make up 80% of the healthcare work force in Canada) (Government of Canada, 2020c; Public Health Agency of Canada, 2019a, b; Canadian Institute for Health Information, 2017). Other public health recommendations to prevent the spread of influenza focus on improving personal hand hygiene, avoiding crowded places through the practice of social distancing, covering coughs and sneezes, and the frequent cleaning or disinfection of common items and surfaces that people touch (Government of Canada, 2020a, 2020b; Infection Prevention and Control Canada, 2020). Recently, these measures have garnered much greater attention as they were applied to control the novel coronavirus outbreak.

COVID-19 is an acute respiratory syndrome caused by the newest member of the family of coronaviruses that has given rise to a recent global pandemic (World Health Organization, 2020a, b, c). The most common symptoms of COVID-19 often resemble those of the common cold and can range in severity from mild to severe (World Health Organization, 2020a, b, c). Both COVID-19 and the influenza virus are primarily respiratory illnesses that are highly transmittable via contact, droplets, and fomites. Thus, public health measures designed to control the spread of COVID-19 may have helped lessen the spread and burden of the seasonal influenza virus. The goal of this study was to compare trends in the 2019–2020 season of influenza in Canada with those of preceding years, and to determine whether any changes in seasonal influenza coincide with specific policies and measures implemented by provincial governments to limit the spread of COVID-19.

## Methods

An ecological study was conducted using information regarding the incident number of influenza cases and associated morbidity and mortality in Canada. Data were obtained from FluNet, the WHO influenza surveillance database, and FluWatch, Canada’s national surveillance system for weekly monitoring of the spread of influenza and other associated illnesses (Benchimol et al., 2015; Government of Canada, 2020a, b). FluWatch relies on a system of laboratories, hospitals, and doctors’ offices to collect data regarding laboratory-confirmed cases of influenza across all provincial or territorial ministries of health. In Canada, the flu season is defined when at least 5% of the influenza tests administered are positive with a minimum of 15 positive tests recorded nationally (Government of Canada, 2021). This often occurs around week 47 of the calendar year (mid-November) with data collection typically beginning in week 35 (Government of Canada, 2021). Key parameters involving data collection help inform policies to limit the spread of influenza. The seven components of influenza surveillance in Canada are: the geographic spread of influenza and influenza-like illness (ILI) activity; laboratory-confirmed detections; syndromic surveillance; outbreak surveillance; severe outcomes surveillance; strain characterization and antiviral resistance testing; and vaccine monitoring (Government of Canada, 2019). Data for FluNet are provided remotely via national influenza centres, national influenza reference laboratories, or WHO regional databases and contain results pertaining to influenza strain characterization (World Health Organization, 2020a, b, c). Information regarding the spread of COVID-19 and subsequent measures implemented across various health regions to limit its spread were obtained from the Government of Canada and Canadian Medical Association Journal respectively (Government of Canada, 2020a, b; Vogel, 2020).

Of particular interest to this study was information characterizing the circulating strains of four influenza subtypes: A (unsubtyped), A (H3N2), A (H1N1), and B, and data classifying the new number of laboratory-confirmed influenza or ILI outbreaks. These data were retrieved from weekly influenza reports across the last four flu seasons (2016–2020) and subsequently separated into three distinct time periods related to the onset of COVID-19 in Canada, and the ensuing policies implemented to limit its spread. Creating the three distinct time frames for each season made it possible to compare the 2019–2020 flu season to the same time periods in preceding years. The first period of the flu season was assigned to epidemiological weeks 41 (2019) to 6 (2020), spanning from the onset of flu reporting in October to the middle of February, prior to the initiation of measures intended to control COVID-19. Weeks 7 to 12 (mid-February to the end of March) of the 2019–2020 flu season correspond to the period at which the majority of the COVID-19-related closures and policies were implemented across Canada. Last, weeks 13 to 18 (end of March to the beginning of May) were chosen to investigate whether COVID-19-related measures had any influence on the incidence of the seasonal influenza during the time frame most often corresponding to the tail end of the flu season. A comprehensive dataset providing weekly accounts documenting the number and subtype of influenza strain was available across four seasons. This provided us with the information necessary to make most of our comparisons. By contrast, there was a lack of consistency in the reporting of ILI across various health regions between 2016 and 2020. Thus, we limited the use of ILI to specific outbreak comparisons between 2016 and 2020.

Data were tabulated in Excel® and analyzed in STATA® version 15.0. Box plots were used to provide a visual comparison of the incidence of influenza cases and outbreaks across designated seasons and time frames. Kruskal-Wallis test was used to compare the incidence and type of influenza cases, as well as the location and number of influenza-associated outbreaks, in order to identify differences across the 4 years for each range of weeks. Then, Wilcoxon rank-sum test was used to perform specific pair-wise comparisons between weeks 13 and 18 in season 2019–2020 and the same weeks in the other seasons. Finally, Kruskal-Wallis test was used to compare weeks 13 to 18 with weeks 41 to 6 and 7 to 12 within the season 2019–2020. A bilateral *p*-value of ≤ 0.05 was considered significant.

## Results

**Fig. 1:**
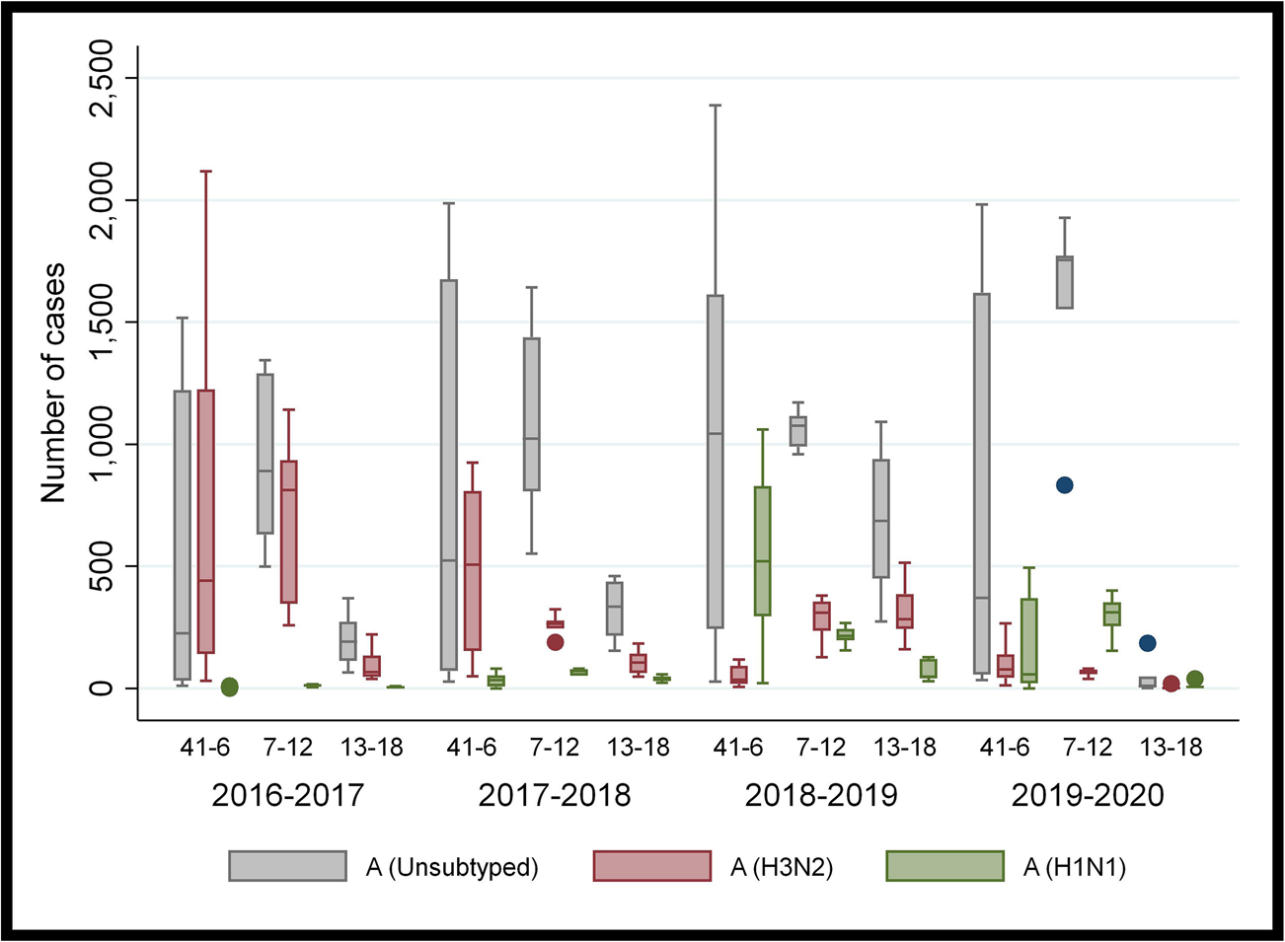
Box plot of the number of positive influenza tests in Canada categorized by influenza A subtype and weekly time frame for four influenza seasons, 2016–2017 to 2019–2020

**Fig. 2:**
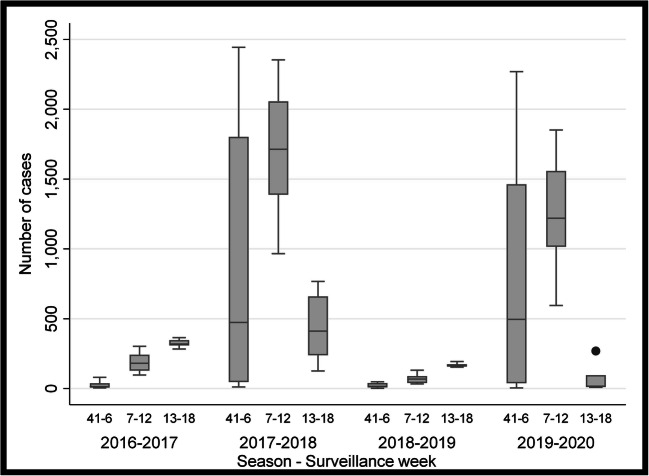
Box plot of the number of positive influenza B tests in Canada divided by weekly time frame for four influenza seasons, 2016–2017 to 2019–2020

Table 1 and Figs. 1 and 2 show the incidence of positive influenza tests in four flu seasons according to influenza subtypes: A (unsubtyped), A (H3N2), A (H1N1), and B. No differences were found for the incidence of influenza A (unsubtyped) over the past four seasons when comparing the time frames of weeks 41 to 6 (*p* = 0.161). From weeks 7 to 12, a significantly higher number of influenza A (unsubtyped) cases were reported in 2020 than in preceding seasons (*p* = 0.0494). However, a significantly lower number of A (unsubtyped) cases were recorded in the final time period from weeks 13 to 18 in 2020 as compared with other seasons (*p* = 0.0007). When comparing the results for influenza A (H3N2), a significantly lower number of influenza cases were documented during the time frames spanning weeks 7 to 12 and 13 to 18 in the 2020 flu season as compared with other seasons (*p* = 0.0010, *p* = 0.0003, respectively) (Table 1 and Fig. 1). Similar findings were reported for influenza B, with the 2020 season revealing a significantly lower number of positive tests between weeks 13 and 18 (*p* = 0.0019) (Fig. 2). Significant differences were also noted in the incidence of the influenza A (H1N1) strain; however, the 2020 season was found to have a significantly higher incidence of cases between weeks 7 and 12 over the preceding seasons (*p* = 0.0003). For each strain during the 2020 flu season, a significantly lower number of positive influenza tests were recorded between weeks 13 and 18 than in the two preceding time frames.

**Table 1:** Number of positive influenza tests in Canada categorized by subtype and weekly time frame for influenza seasons, 2016–2017 to 2019–2020

Influenza type Weeks	2016–2017 Median (P_25_–P_75_)	2017–2018 Median (P_25_–P_75_)	2018–2019 Median (P_25_–P_75_)	2019–2020 Median (P_25_–P_75_)	*p*-value*
A (unsubtyped)					
41–6	226.5 (31–1222)	523 (71–1675)	1042.5 (244–1613)	372 (57–1620)	0.161
7–12	889.5 (629–1291)	1023 (807–1437)	1075.5 (990–1116)	1754.5 (1552–1772)	0.049
13–18	191.5 (113–273)	336 (215–436)	685 (448–939)	9.5 (4–48)	0.0007
A H3N2					
41–6	440.5 (140–1225)	506 (153–808)	35 (18–93)	77 (43–139)	0.0001
7–12	812.5 (347–935)	265 (246–279)	310 (236–357)	72 (58–76)	0.001
13–18	66 (47–134)	105.5 (62–143)	284.5 (244–387)	1 (0–5)	0.0003
A H1N1					
41–6	4 (4–4)	33 (7–54)	519.5 (295–828)	57.5 (20–370)	0.0001
7–12	12 (7–14)	73 (51–76)	214.5 (197–244)	312 (255–354)	0.0003
13–18	4 (0–6)	37 (29–49)	48.5 (41–121)	5 (0–11)	0.001
B					
41–6	14.5 (7–39)	473 (47–1802)	15.5 (8 – 40)	495 (39–1463)	0.0001
7–12	181 (128–242)	1714 (1387–2057)	67.5 (40 – 90)	1219.5 (1015–1599)	0.0003
13–18	318.5 (309–347)	411 (238–661)	168 (157–175)	16.5 (11–97)	0.0019
Total influenza cases	37,862	63,405	46,662	53,783	

*P*, percentile. *Kruskal-Wallis test was used to identify differences across the 4 years, and for each of the 3 periods within each season (weeks 41–6, 7–12, 13–18)

The effects of measures implemented in weeks 13 to 18 in 2020 compared with previous time frames and seasons are further quantified in Table 2. This analysis was performed using an incidence proportion estimated as the total number of cases for the surveillance week for each season divided by the total population of Canada reported per quarterly for 2016 to 2020, and multiplied by 100,000 (Statistics Canada, 2020). We then compared the incidence proportion in weeks 41–6 vs 13–18, and 7–12 vs 13–18, and the incidence proportion in weeks 13–18 in season 2016–2017 vs 2019–2020, 2017–2018 vs 2019–2020, and 2018–2019 vs 2019–2020 using chi-squared analysis. All comparisons reported a statistically significant decrease in the proportion of influenza cases recorded in weeks 13 to 18 in 2020 compared with previous time frames and seasons (*p* < 0.0001).

**Table 2:** Incidence proportion of laboratory-confirmed influenza cases in Canada between 2016 and 2020

Surveillance week	Influenza cases				Incidence proportion (new cases/100,000 population)			
	2016–2017	2017–2018	2018–2019	2019–2020	2016–2017	2017–2018	2018–2019	2019–2020
41–6	23,077	42,405	29,403	33,786	63	115	79	89*
7–12	11,046	15,573	9831	19,235	30	42	26	51*
13–18	3739	5427	7428	762	10**	15**	20**	2
Total	37,862	63,405	46,662	53,783	104	172	125	142

*p < 0.0001. Comparison of the incidence proportion in weeks 41–6 vs 13–18, and 7–12 vs 13–18

**p < 0.0001. Comparison of the incidence proportion in weeks 13–18 in seasons 2016–2017, 2017–2018, and 2018–2019 vs 2019–2020

**Fig. 3:**
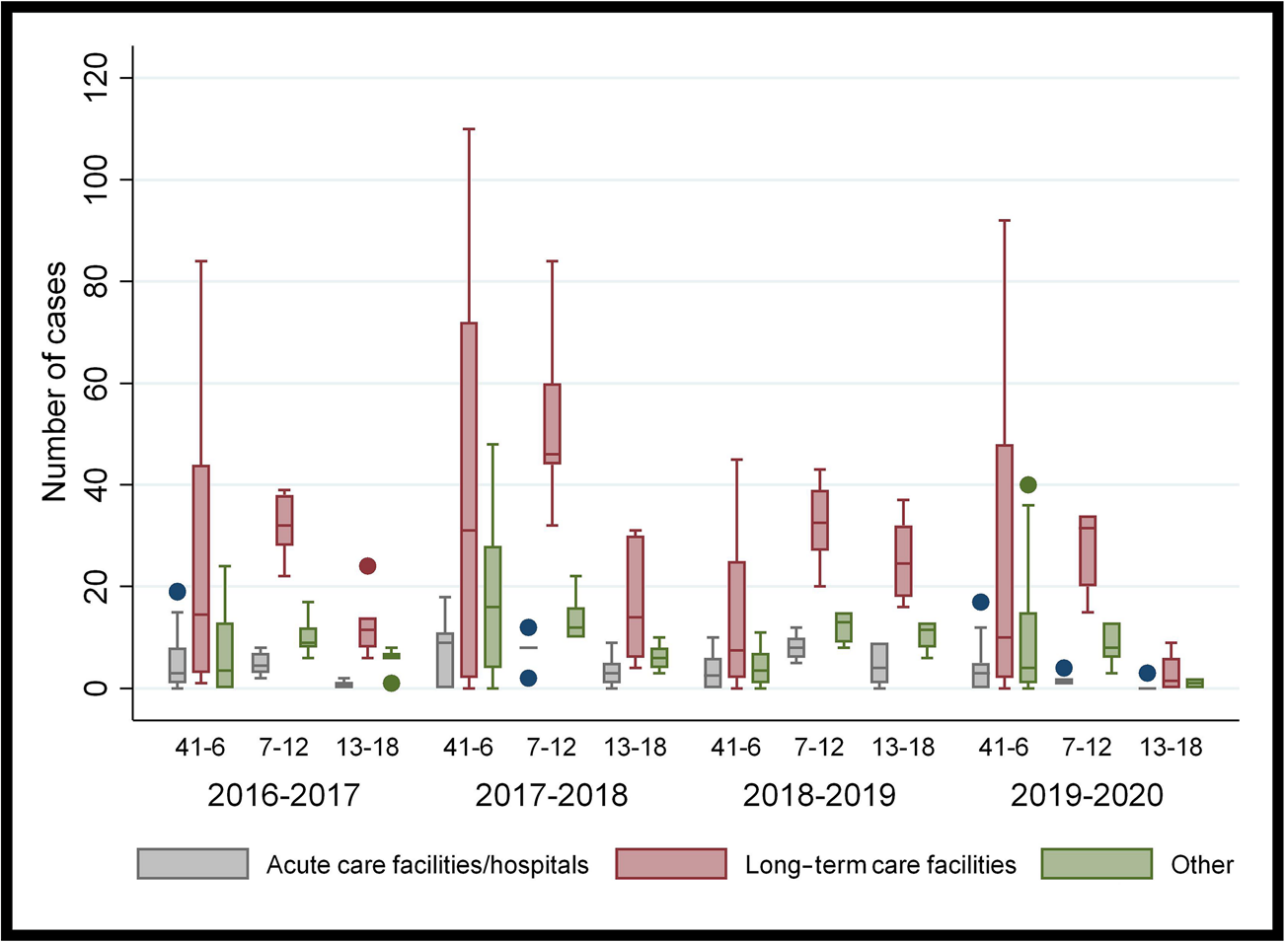
Box plot of the number and location of new influenza outbreaks in Canada by weekly time frame for four influenza seasons, 2016–2017 to 2019–2020

Analysis was also undertaken to assess the number and location of new laboratory-confirmed influenza cases by week (Table 3 and Fig. 3). No significant differences were recorded in the number of outbreaks occurring in hospitals or long-term care facilities between weeks 41 and 6 of 2019–2020 compared with the three preceding flu seasons. However, during weeks 7–12, a significantly lower number of hospital-associated outbreaks were reported in 2020 than in the preceding seasons (*p* = 0.0042). Similar results were also observed for weeks 13–18 in both long-term care facilities and facilities classified as other (*p* = 0.0035, *p* = 0.0014, respectively). Last, the number of outbreaks in 2020 at each type of facility was significantly lower during weeks 13 to 18 than during the two preceding time frames. Supplementary material S1 has the output of the statistical analysis for specific pair-wise comparisons.

**Table 3:** Number and location of new influenza outbreaks in Canada by weekly time frame for four influenza seasons, 2016–2017 to 2019–2020

Setting Weeks	2016–2017 Median (P_25_–P_75_)	2017–2018 Median (P_25_–P_75_)	2018–2019 Median (P_25_–P_75_)	2019–2020 Median (P_25_–P_75_)	*p*-value*
Hospitals					
41–6	3 (1–8)	9 (0–11)	2.5 (0–6)	3 (0–5)	0.309
7–12	4.5 (3–7)	8 (8–8)	8 (6–10)	1 (1–2)	0.004
13–18	1 (0–1)	3 (1–5)	4 (1–9)	0 (0–0)	0.046
Long-term care facilities					
41–6	14.5 (3–44)	31 ( 2–72)	7.5 (2–25)	10 (2–48)	0.475
7–12	32 (28–38)	46 (44–60)	32.5 (27–39)	31.5 (20–34)	0.048
13–18	11.5 (8–14)	14 (6–30)	24.5 (18–32)	1.5 (0–6)	0.003
Other					
41–6	3.5 (0–13)	16 (4–28)	3.5 (1–7)	4 (1–15)	0.034
7–12	9 (8–12)	12 (10–16)	13 (9–15)	8 (6–13)	0.159
13–18	6 (6–7)	6 (4–8)	11.5 (8–13)	1 (0–2)	0.001

*P*, percentile. *Kruskal-Wallis test was used to identify differences across the 4 years, and for each of the 3 periods within each season (weeks 41–6, 7–12, 13–18)

## Discussion

**Fig. 4:**
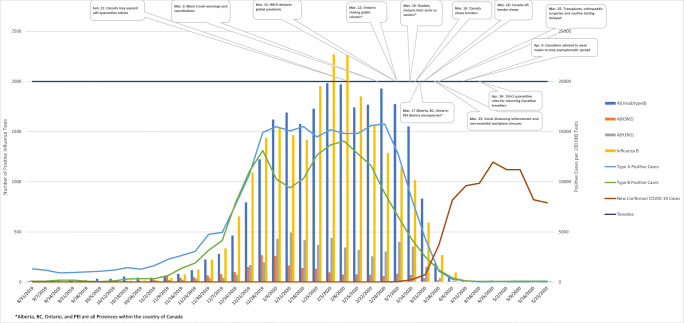
Incidence of influenza and COVID-19 by week in Canada in relation to the implementation of control measures to limit the spread of COVID-19

In the present study, a significantly lower number of influenza cases were reported in Canada in 2020 between weeks 7 and 18 (February 9, 2020 to May 1, 2020) for influenza A (H3N2) and B viruses as compared with previous years. A high number of cases caused by influenza A (unsubtyped) were reported in weeks 7–12, with a precipitous decline during weeks 13 to 18 (March 22, 2020 to May 1, 2020). The weeks during which fewer cases were reported roughly correspond to the implementation of social distancing measures to control the spread of COVID-19 in Canada. As depicted in Fig. 4, most of the initial warnings related to travel occurred near the middle of February, around week 7. This was then followed by the enactment of policies designed to prevent human to human transmission of the virus towards the middle of March. For example, on March 16, 2020, Canada made the decision to close its border to all non-citizens or non-permanent residents with exceptions being made for essential workers and citizens of the United States. On March 18, 2020, Canada and the USA mutually agreed to close their shared border to all non-essential travel indefinitely. In addition, on March 23, 2020, provinces such as Ontario and Quebec began to implement policies ordering for the temporary closure of all non-essential workplaces (Vogel, 2020). Thus, the lower number of cases reported for influenza strains A (H3N2) and B could reflect the initial warnings related to COVID, while the lower incidence of cases following week 13 may be more reflective of the actual policies implemented. Kuo et al. observed similar trends in Taiwan (Kuo et al., 2020). This theory is further substantiated by the fact that the number of influenza cases reported in 2020 for each strain was significantly lower during weeks 13 to 18 than during either of the previously recorded time frames spanning from weeks 41 to 12. As a result, it was determined that the 2020 flu season ended abruptly in week 12, approximately 2 months earlier than in each of the past three seasons. This change in course could be the result of interrupted influenza reporting as several countries around the globe have had to change their focus or repurpose their influenza surveillance systems to also track the COVID-19 virus (World Health Organization, 2020a, b, c; Government of Canada, 2020a, b). However, reports by the WHO suggest that the number of influenza tests administered in Canada (test numbers are not included in FluWatch reports) actually increased prior to and during the onset of COVID-19 (World Health Organization, 2020a, b, c). In fact, the time period at which the incidence of influenza cases decreased most significantly was the same period during which the most tests were processed.

The number of laboratory-documented outbreaks also appeared to be related to the onset of COVID-19 measures. Significantly lower numbers of outbreaks in several types of facilities were documented in 2020 as compared with previous seasons. In addition, the number of outbreaks reported in each facility was found to be significantly less between weeks 13 and 18 than in the previously reported time periods. These findings correspond to the approximate dates at which key closures or restrictions were implemented by various levels of government (Fig. 4). For example, the closure of public schools in the province of Ontario and the restrictions imposed on visiting seniors in long-term care facilities in two provinces, Ontario and Quebec, align with decreased documented influenza outbreaks in those particular settings. While the exact effect of social distancing measures on influenza outbreaks is difficult to quantify, it would be useful to have a better understanding about how human behaviours could be modified to more effectively protect those who are vulnerable.

This study is not without limitations. First, Canada’s geography makes it difficult to conduct influenza surveillance, as the timing and burden of influenza activity differ considerably across the country (Public Health Agency of Canada, 2019a, b). Health ministries and hospitals that participate in FluWatch vary in the information collected and recorded and the heterogeneity in flu surveillance data presents challenges to having a comprehensive and representative dataset for analysis. Furthermore, FluWatch data are not disaggregated by sex and age, which limits our analyses to generalizations across the population, although there may have been quite different results within population groups. High variability between annual influenza seasons makes comparison difficult. We endeavoured to overcome this limitation by performing the comparison across several influenza seasons, and incorporate weeks 41 to 6 into our analysis, before the first COVID-19 case diagnosed in Canada (Marchand-Senécal et al., 2020). Additionally, many people with mild to moderate illnesses or ILI may have avoided clinics, thus limiting the number of influenza tests ordered and documented. This would have been especially true during the first peak of the COVID-19 pandemic when Canadians were strongly encouraged to self-isolate and remain socially distant.

## Conclusion

The measures and policies that were implemented in Canada to limit the spread of COVID-19 appear to have led to a corresponding decrease in the incidence of seasonal influenza virus. However, specific policies that were effective in controlling the pandemic, for example social gathering and travel restrictions, are unlikely to be implemented annually. In contrast, more simple behaviours related to social distancing, limited gathering sizes, and improving personal hygiene are still effective in controlling the spread of influenza and may also be more readily adopted (Chiu et al., 2020; Fong et al., 2020). Ultimately, further research will be necessary to determine how we can effectively use this information to inform policies and behaviours that lessen the burden of seasonal influenza for Canadians and upon our healthcare system.

### Contributions to knowledge

What does this study add to existing knowledge?

- This study aimed to study the effectiveness of specific policies and measures that were implemented to control the COVID-19 pandemic. The knowledge gained provides insight into specific behaviours that are effective in controlling the spread of influenza and serve as the basis for future research that helps us lessen the burden of seasonal influenza going forward.
  What are the key implications for public health interventions, practice or policy?
- Key implications for public health policy that can be taken from this study suggest that relatively simple behaviours are effective at lessening the spread of influenza. Public health interventions endorsing personal hygiene or social distancing may serve as effective means by which we can reduce the spread of transmissible viruses in the future.

## Supplementary Information

ESM 1 (PDF 238 kb)

## Data Availability

Analyses available upon request
