# Supplementary material for: Changes in the incidence of seasonal influenza in response to COVID-19 social distancing measures: an observational study based on Canada’s national influenza surveillance system
Source: Can J Public Health. 2021 May 28;112(4):620–8. doi: 10.17269/s41997-021-00509-4 (PMC8161713; doi:10.17269/s41997-021-00509-4)
Supplement: Supplementary file 1 — (PDF 238 kb) [file 41997_2021_509_MOESM1_ESM.pdf]

Output of the program
Comparison of season for each week:

```
. kwallis A_uns if week ==0, by(season)

Kruskal-Wallis equality-of-populations rank test

  +---------------------------+
  |    season | Obs | Rank Sum |
  |-----------+-----+----------|
  | 2016-2017 |  18 |   505.00 |
  | 2017-2018 |  19 |   740.50 |
  | 2018-2019 |  18 |   786.50 |
  | 2019-2020 |  18 |   669.00 |
  +---------------------------+

chi-squared =     5.156 with 3 d.f.
probability =     0.1607

chi-squared with ties =     5.157 with 3 d.f.
probability =     0.1607

. kwallis A_uns if week ==1, by(season)

Kruskal-Wallis equality-of-populations rank test

  +---------------------------+
  |    season | Obs | Rank Sum |
  |-----------+-----+----------|
  | 2016-2017 |   6 |    47.00 |
  | 2017-2018 |   5 |    53.00 |
  | 2018-2019 |   6 |    66.00 |
  | 2019-2020 |   6 |   110.00 |
  +---------------------------+

chi-squared =     7.840 with 3 d.f.
probability =     0.0494

chi-squared with ties =     7.840 with 3 d.f.
probability =     0.0494

. kwallis A_uns if week ==2, by(season)

Kruskal-Wallis equality-of-populations rank test

  +---------------------------+
  |    season | Obs | Rank Sum |
  |-----------+-----+----------|
  | 2016-2017 |   6 |    63.00 |
  | 2017-2018 |   6 |    89.00 |
  | 2018-2019 |   6 |   123.00 |
  | 2019-2020 |   6 |    25.00 |
  +---------------------------+

chi-squared =    17.147 with 3 d.f.
probability =     0.0007
```

```
chi-squared with ties =    17.147 with 3 d.f.
probability =     0.0007
```

EN LA ULTIMA SEMANA COMPROBAR SI LA ULTIMA SEASON ES LA QUE DIFIERE DE LAS OTRAS

```
. ranksum A_uns if week ==2  & (season ==1| season ==4)  , by(season)

Two-sample Wilcoxon rank-sum (Mann-Whitney) test

      season |      obs    rank sum    expected
-------------+---------------------------------
   2016-2017 |        6          54          39
   2019-2020 |        6          24          39
-------------+---------------------------------
    combined |       12          78          78

unadjusted variance       39.00
adjustment for ties        0.00
                     ----------
adjusted variance         39.00

Ho: A_uns(season==2016-2017) = A_uns(season==2019-2020)
             z =   2.402
    Prob > |z| =   0.0163

. ranksum A_uns if week ==2  &   (season ==2| season ==4)  , by(season)

Two-sample Wilcoxon rank-sum (Mann-Whitney) test

      season |      obs    rank sum    expected
-------------+---------------------------------
   2017-2018 |        6          56          39
   2019-2020 |        6          22          39
-------------+---------------------------------
    combined |       12          78          78

unadjusted variance       39.00
adjustment for ties        0.00
                     ----------
adjusted variance         39.00

Ho: A_uns(season==2017-2018) = A_uns(season==2019-2020)
             z =   2.722
    Prob > |z| =   0.0065

. ranksum A_uns if week ==2  &   (season ==3| season ==4)  , by(season)

Two-sample Wilcoxon rank-sum (Mann-Whitney) test

      season |      obs    rank sum    expected
-------------+---------------------------------
   2018-2019 |        6          57          39
   2019-2020 |        6          21          39
-------------+---------------------------------
```

```
    combined |       12          78          78

unadjusted variance       39.00
adjustment for ties        0.00
                     ----------
adjusted variance         39.00

Ho: A_uns(season==2018-2019) = A_uns(season==2019-2020)
            z =   2.882
    Prob > |z| =   0.0039
```

EN LA ULTIMA SEASON COMPARAR LAS TRES SEMANAS

```
. kwallis A_uns if season ==4, by(week)

Kruskal-Wallis equality-of-populations rank test

  +------------------------+
  |  week | Obs | Rank Sum |
  |-------+-----+----------|
  |  41-6 |  18 |   290.00 |
  |  7-12 |   6 |   144.00 |
  | 13-18 |   6 |    31.00 |
  +------------------------+

chi-squared =    13.947 with 2 d.f.
probability =     0.0009

chi-squared with ties =    13.947 with 2 d.f.
probability =     0.0009
```

A H3N2

COMPARACIÓN DE LAS ESTACIONES SEGÚN SEMANA

```
. kwallis A_H3N2 if week ==0, by(season)

Kruskal-Wallis equality-of-populations rank test

  +-------------------------+
  | season | Obs | Rank Sum |
  |--------+-----+----------|
  |      1 |  18 |   933.00 |
  |      2 |  19 |   971.50 |
  |      3 |  18 |   311.50 |
  |      4 |  18 |   485.00 |
  +-------------------------+

chi-squared =    36.779 with 3 d.f.
probability =     0.0001

chi-squared with ties =    36.783 with 3 d.f.
probability =     0.0001
```

```
kwallis A_H3N2 if week ==1, by(season)

Kruskal-Wallis equality-of-populations rank test

  +-------------------------+
  | season | Obs | Rank Sum |
  |--------+-----+----------|
  |      1 |   6 |   114.00 |
  |      2 |   5 |    58.00 |
  |      3 |   6 |    83.00 |
  |      4 |   6 |    21.00 |
  +-------------------------+

chi-squared =    16.271 with 3 d.f.
probability =     0.0010

chi-squared with ties =    16.271 with 3 d.f.
probability =     0.0010

. . kwallis A_H3N2 if week ==2, by(season)

Kruskal-Wallis equality-of-populations rank test

  +-------------------------+
  | season | Obs | Rank Sum |
  |--------+-----+----------|
  |      1 |   6 |    71.50 |
  |      2 |   6 |    80.50 |
  |      3 |   6 |   127.00 |
  |      4 |   6 |    21.00 |
  +-------------------------+

chi-squared =    18.875 with 3 d.f.
probability =     0.0003

chi-squared with ties =    18.900 with 3 d.f.
probability =     0.0003
```

EN LA ULTIMA SEMANA COMPROBAR SI LA ULTIMA SEASON ES LA QUE DIFIERE DE LAS OTRAS

```
. ranksum A_H3N2 if week ==2  & (season ==1| season ==4)  , by(season)

Two-sample Wilcoxon rank-sum (Mann-Whitney) test

      season |      obs    rank sum    expected
-------------+---------------------------------
           1 |        6          57          39
           4 |        6          21          39
-------------+---------------------------------
    combined |       12          78          78

unadjusted variance       39.00
adjustment for ties       -0.27
                     ----------
adjusted variance         38.73
```

```
Ho: A_H3N2(season==1) = A_H3N2(season==4)
             z =   2.892
    Prob > |z| =   0.0038

. . ranksum A_H3N2 if week ==2  &   (season ==2| season ==4)  ,
by(season)

Two-sample Wilcoxon rank-sum (Mann-Whitney) test

      season |      obs    rank sum    expected
-------------+---------------------------------
           2 |        6          57          39
           4 |        6          21          39
-------------+---------------------------------
    combined |       12          78          78

unadjusted variance       39.00
adjustment for ties       -0.27
                     ----------
adjusted variance         38.73

Ho: A_H3N2(season==2) = A_H3N2(season==4)
             z =   2.892
    Prob > |z| =   0.0038

. ranksum A_H3N2 if week ==2  &   (season ==3| season ==4)  , by(season)

Two-sample Wilcoxon rank-sum (Mann-Whitney) test

      season |      obs    rank sum    expected
-------------+---------------------------------
           3 |        6          57          39
           4 |        6          21          39
-------------+---------------------------------
    combined |       12          78          78

unadjusted variance       39.00
adjustment for ties       -0.27
                     ----------
adjusted variance         38.73

Ho: A_H3N2(season==3) = A_H3N2(season==4)
             z =   2.892
    Prob > |z| =   0.0038
```

EN LA ULTIMA SEASON COMPARAR LAS TRES SEMANAS

```
Kruskal-Wallis equality-of-populations rank test

  +-------------------------+
  |  week | Obs | Rank Sum |
  |-------+-----+----------|
  |  41-6 |  18 |   290.00 |
  |  7-12 |   6 |   144.00 |
  | 13-18 |   6 |    31.00 |
```

```
  +-------------------------+

chi-squared =    13.947 with 2 d.f.
probability =     0.0009

chi-squared with ties =    13.947 with 2 d.f.
probability =     0.0009
```

A H1N1

COMPARACIÓN DE LAS ESTACIONES SEGÚN SEMANA

```
kwallis A_H1N1 if week ==0, by(season)

Kruskal-Wallis equality-of-populations rank test

  +-------------------------+
  | season | Obs | Rank Sum |
  |--------+-----+----------|
  |      1 |  18 |   269.00 |
  |      2 |  19 |   589.50 |
  |      3 |  18 |  1093.00 |
  |      4 |  18 |   749.50 |
  +-------------------------+

chi-squared =    44.319 with 3 d.f.
probability =     0.0001

chi-squared with ties =    44.540 with 3 d.f.
probability =     0.0001

. . kwallis A_H1N1 if week ==1, by(season)

Kruskal-Wallis equality-of-populations rank test

  +-------------------------+
  | season | Obs | Rank Sum |
  |--------+-----+----------|
  |      1 |   6 |    21.00 |
  |      2 |   5 |    45.00 |
  |      3 |   6 |    94.00 |
  |      4 |   6 |   116.00 |
  +-------------------------+

chi-squared =    19.170 with 3 d.f.
probability =     0.0003

chi-squared with ties =    19.180 with 3 d.f.
probability =     0.0003

. . kwallis A_H1N1 if week ==2, by(season)

Kruskal-Wallis equality-of-populations rank test
```

```
  +-------------------------+
  | season | Obs | Rank Sum |
  |--------+-----+----------|
  |      1 |   6 |    36.00 |
  |      2 |   6 |    99.50 |
  |      3 |   6 |   118.50 |
  |      4 |   6 |    46.00 |
  +-------------------------+

chi-squared =    16.182 with 3 d.f.
probability =     0.0010

chi-squared with ties =    16.267 with 3 d.f.
probability =     0.0010
```

EN LA ULTIMA SEMANA COMPROBAR SI LA ULTIMA SEASON ES LA QUE DIFIERE DE LAS OTRAS

```
. ranksum A_H1N1 if week ==2 & (season ==1| season ==4)  , by(season)

Two-sample Wilcoxon rank-sum (Mann-Whitney) test

      season |      obs    rank sum    expected
-------------+---------------------------------
   2016-2017 |        6          36          39
   2019-2020 |        6          42          39
-------------+---------------------------------
    combined |       12          78          78

unadjusted variance       39.00
adjustment for ties       -1.50
                     ----------
adjusted variance         37.50

Ho: A_H1N1(season==2016-2017) = A_H1N1(season==2019-2020)
             z =  -0.490
    Prob > |z| =   0.6242


.ranksum A_H1N1 if week ==2  &   (season ==2| season ==4)  , by(season)

Two-sample Wilcoxon rank-sum (Mann-Whitney) test

      season |      obs    rank sum    expected
-------------+---------------------------------
   2017-2018 |        6          54          39
   2019-2020 |        6          24          39
-------------+---------------------------------
    combined |       12          78          78

unadjusted variance       39.00
adjustment for ties       -0.14
                     ----------
adjusted variance         38.86
```

```
Ho: A_H1N1(season==2017-2018) = A_H1N1(season==2019-2020)
             z =   2.406
    Prob > |z| =   0.0161

. . ranksum A_H1N1 if week ==2  &   (season ==3| season ==4)  ,
by(season)

Two-sample Wilcoxon rank-sum (Mann-Whitney) test

      season |      obs    rank sum    expected
-------------+---------------------------------
   2018-2019 |        6          56          39
   2019-2020 |        6          22          39
-------------+---------------------------------
    combined |       12          78          78

unadjusted variance       39.00
adjustment for ties       -0.14
                     ----------
adjusted variance         38.86

Ho: A_H1N1(season==2018-2019) = A_H1N1(season==2019-2020)
             z =   2.727
    Prob > |z| =   0.0064
.
```

EN LA ULTIMA SEASON COMPARAR LAS TRES SEMANAS

```
. kwallis A_H1N1 if season ==4, by(week)

Kruskal-Wallis equality-of-populations rank test

  +-------------------------+
  |  week | Obs | Rank Sum |
  |-------+-----+----------|
  |  41-6 |  18 |   298.50 |
  |  7-12 |   6 |   130.00 |
  | 13-18 |   6 |    36.50 |
  +-------------------------+

chi-squared =    10.082 with 2 d.f.
probability =     0.0065

chi-squared with ties =    10.093 with 2 d.f.
probability =     0.0064
```

B

COMPARACIÓN DE LAS ESTACIONES SEGÚN SEMANA

```
. kwallis B if week ==0, by(season)

Kruskal-Wallis equality-of-populations rank test

  +-----------------------------+
  |    season | Obs | Rank Sum |
  |-----------+-----+----------|
  | 2016-2017 |  18 |   426.50 |
  | 2017-2018 |  19 |   984.50 |
  | 2018-2019 |  18 |   406.00 |
  | 2019-2020 |  18 |   884.00 |
  +-----------------------------+

chi-squared =    30.551 with 3 d.f.
probability =     0.0001

chi-squared with ties =    30.562 with 3 d.f.
probability =     0.0001

. . kwallis B if week ==1, by(season)

Kruskal-Wallis equality-of-populations rank test

  +-----------------------------+
  |    season | Obs | Rank Sum |
  |-----------+-----+----------|
  | 2016-2017 |   6 |    55.00 |
  | 2017-2018 |   5 |    97.00 |
  | 2018-2019 |   6 |    23.00 |
  | 2019-2020 |   6 |   101.00 |
  +-----------------------------+

chi-squared =    18.746 with 3 d.f.
probability =     0.0003

chi-squared with ties =    18.746 with 3 d.f.
probability =     0.0003

. . kwallis B if week ==2, by(season)

Kruskal-Wallis equality-of-populations rank test

  +-----------------------------+
  |    season | Obs | Rank Sum |
  |-----------+-----+----------|
  | 2016-2017 |   6 |   107.00 |
  | 2017-2018 |   6 |   107.00 |
  | 2018-2019 |   6 |    57.00 |
  | 2019-2020 |   6 |    29.00 |
  +-----------------------------+

chi-squared =    14.960 with 3 d.f.
probability =     0.0019

chi-squared with ties =    14.960 with 3 d.f.
probability =     0.0019
```

EN LA ULTIMA SEMANA COMPROBAR SI LA ULTIMA SEASON ES LA QUE DIFIERE DE LAS OTRAS

```
. ranksum B if week ==2  & (season ==1| season ==4)  , by(season)

Two-sample Wilcoxon rank-sum (Mann-Whitney) test

      season |      obs    rank sum    expected
-------------+---------------------------------
   2016-2017 |        6          57          39
   2019-2020 |        6          21          39
-------------+---------------------------------
    combined |       12          78          78

unadjusted variance       39.00
adjustment for ties        0.00
                     ----------
adjusted variance         39.00

Ho: B(season==2016-2017) = B(season==2019-2020)
             z =   2.882
    Prob > |z| =   0.0039

. . ranksum B if week ==2  &   (season ==2| season ==4)  , by(season)

Two-sample Wilcoxon rank-sum (Mann-Whitney) test

      season |      obs    rank sum    expected
-------------+---------------------------------
   2017-2018 |        6          55          39
   2019-2020 |        6          23          39
-------------+---------------------------------
    combined |       12          78          78

unadjusted variance       39.00
adjustment for ties        0.00
                     ----------
adjusted variance         39.00

Ho: B(season==2017-2018) = B(season==2019-2020)
             z =   2.562
    Prob > |z| =   0.0104

. . ranksum B if week ==2  &   (season ==3| season ==4)  , by(season)

Two-sample Wilcoxon rank-sum (Mann-Whitney) test

      season |      obs    rank sum    expected
-------------+---------------------------------
   2018-2019 |        6          51          39
   2019-2020 |        6          27          39
-------------+---------------------------------
    combined |       12          78          78

unadjusted variance       39.00
adjustment for ties        0.00
                     ----------
```

```
adjusted variance         39.00

Ho: B(season==2018-2019) = B(season==2019-2020)
            z =   1.922
    Prob > |z| =   0.0547
```

EN LA ULTIMA SEASON COMPARAR LAS TRES SEMANAS

```
. kwallis B if season ==4, by(week)

Kruskal-Wallis equality-of-populations rank test

  +------------------------+
  |  week | Obs | Rank Sum |
  |-------+-----+----------|
  |  41-6 |  18 |   294.50 |
  |  7-12 |   6 |   128.00 |
  | 13-18 |   6 |    42.50 |
  +------------------------+

chi-squared =     8.291 with 2 d.f.
probability =     0.0158

chi-squared with ties =     8.295 with 2 d.f.
probability =     0.0158
```

LAB_DX_FLU_PLACE

ACUTE CARE FACILITIES/HOSPITALS

COMPARACIÓN DE LAS ESTACIONES SEGÚN SEMANA

```
. kwallis hospitals if week ==0, by(season)

Kruskal-Wallis equality-of-populations rank test

  +----------------------------+
  |    season | Obs | Rank Sum |
  |-----------+-----+----------|
  | 2016-2017 |  18 |   679.00 |
  | 2017-2018 |  19 |   836.00 |
  | 2018-2019 |  18 |   572.00 |
  | 2019-2020 |  18 |   614.00 |
  +----------------------------+

chi-squared =     3.513 with 3 d.f.
probability =     0.3191
```

```
chi-squared with ties =     3.587 with 3 d.f.
probability =     0.3096

. kwallis hospitals if week ==1, by(season)

Kruskal-Wallis equality-of-populations rank test

  +-----------------------------+
  |    season | Obs | Rank Sum |
  |-----------+-----+----------|
  | 2016-2017 |   6 |    67.00 |
  | 2017-2018 |   5 |    81.00 |
  | 2018-2019 |   6 |   102.50 |
  | 2019-2020 |   6 |    25.50 |
  +-----------------------------+

chi-squared =    13.213 with 3 d.f.
probability =     0.0042

chi-squared with ties =    13.398 with 3 d.f.
probability =     0.0039

. kwallis hospitals if week ==2, by(season)

Kruskal-Wallis equality-of-populations rank test

  +-----------------------------+
  |    season | Obs | Rank Sum |
  |-----------+-----+----------|
  | 2016-2017 |   6 |    61.50 |
  | 2017-2018 |   6 |    96.00 |
  | 2018-2019 |   6 |    99.50 |
  | 2019-2020 |   6 |    43.00 |
  +-----------------------------+

chi-squared =     7.492 with 3 d.f.
probability =     0.0578

chi-squared with ties =     8.011 with 3 d.f.
probability =     0.0458
```

EN LA ULTIMA SEMANA COMPROBAR SI LA ULTIMA SEASON ES LA QUE DIFIERE DE LAS OTRAS

```
. ranksum hospitals if week ==2  & (season ==1| season ==4)  , by(season)

Two-sample Wilcoxon rank-sum (Mann-Whitney) test

      season |      obs    rank sum    expected
-------------+---------------------------------
   2016-2017 |        6          46          39
   2019-2020 |        6          32          39
-------------+---------------------------------
    combined |       12          78          78

unadjusted variance       39.00
```

```
adjustment for ties       -8.18
                     ----------
adjusted variance         30.82

Ho: hospit~s(season==2016-2017) = hospit~s(season==2019-2020)
             z =   1.261
    Prob > |z| =   0.2073

. . ranksum hospitals if week ==2  &   (season ==2| season ==4)  ,
by(season)

Two-sample Wilcoxon rank-sum (Mann-Whitney) test

      season |      obs    rank sum    expected
-------------+---------------------------------
   2017-2018 |        6        51.5          39
   2019-2020 |        6        26.5          39
-------------+---------------------------------
    combined |       12          78          78

unadjusted variance       39.00
adjustment for ties       -5.32
                     ----------
adjusted variance         33.68

Ho: hospit~s(season==2017-2018) = hospit~s(season==2019-2020)
             z =   2.154
    Prob > |z| =   0.0313

. . ranksum hospitals if week ==2  &   (season ==3| season ==4)  ,
by(season)

Two-sample Wilcoxon rank-sum (Mann-Whitney) test

      season |      obs    rank sum    expected
-------------+---------------------------------
   2018-2019 |        6        51.5          39
   2019-2020 |        6        26.5          39
-------------+---------------------------------
    combined |       12          78          78

unadjusted variance       39.00
adjustment for ties       -4.91
                     ----------
adjusted variance         34.09

Ho: hospit~s(season==2018-2019) = hospit~s(season==2019-2020)
             z =   2.141
    Prob > |z| =   0.0323
```

EN LA ULTIMA SEASON COMPARAR LAS TRES SEMANAS

```
. . kwallis hospitals if season ==4, by(week)

Kruskal-Wallis equality-of-populations rank test
```

```
  +-------------------------+
  |  week | Obs | Rank Sum |
  |-------+-----+----------|
  |  41-6 |  18 |   320.50 |
  |  7-12 |   6 |    97.00 |
  | 13-18 |   6 |    47.50 |
  +-------------------------+

chi-squared =     5.721 with 2 d.f.
probability =     0.0572

chi-squared with ties =     6.034 with 2 d.f.
probability =     0.0489
```

LONG TERM CARE FACILITIES

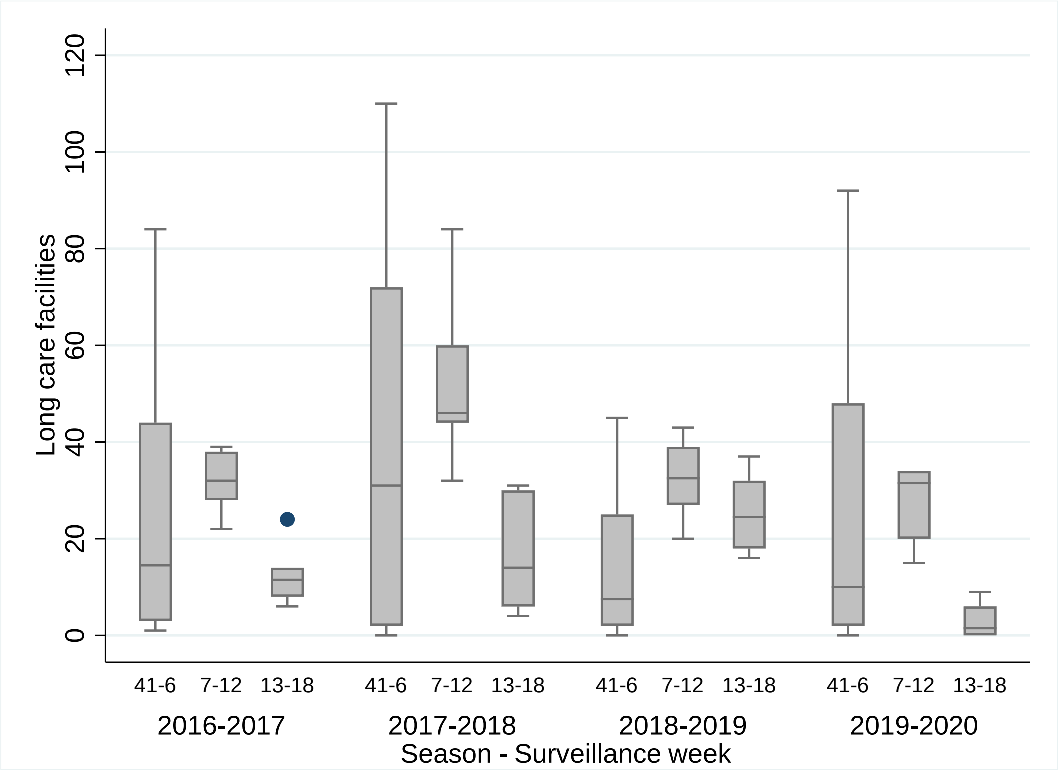

COMPARACIÓN DE LAS ESTACIONES SEGÚN SEMANA

```
. . kwallis long_term_fac if week ==0, by(season)

Kruskal-Wallis equality-of-populations rank test

  +-----------------------------+
  |    season | Obs | Rank Sum |
  |-----------+-----+----------|
  | 2016-2017 |  18 |   721.00 |
  | 2017-2018 |  19 |   785.00 |
  | 2018-2019 |  18 |   571.00 |
```

```
  | 2019-2020 |  18 |   624.00 |
  +----------------------------+

chi-squared =     2.491 with 3 d.f.
probability =     0.4769

chi-squared with ties =     2.500 with 3 d.f.
probability =     0.4753

. . kwallis long_term_fac if week ==1, by(season)

Kruskal-Wallis equality-of-populations rank test

  +----------------------------+
  |    season | Obs | Rank Sum |
  |-----------+-----+----------|
  | 2016-2017 |   6 |    63.50 |
  | 2017-2018 |   5 |    96.00 |
  | 2018-2019 |   6 |    67.00 |
  | 2019-2020 |   6 |    49.50 |
  +----------------------------+

chi-squared =     7.821 with 3 d.f.
probability =     0.0499

chi-squared with ties =     7.911 with 3 d.f.
probability =     0.0479

. . kwallis long_term_fac if week ==2, by(season)

Kruskal-Wallis equality-of-populations rank test

  +----------------------------+
  |    season | Obs | Rank Sum |
  |-----------+-----+----------|
  | 2016-2017 |   6 |    74.00 |
  | 2017-2018 |   6 |    82.50 |
  | 2018-2019 |   6 |   116.50 |
  | 2019-2020 |   6 |    27.00 |
  +----------------------------+

chi-squared =    13.612 with 3 d.f.
probability =     0.0035

chi-squared with ties =    13.665 with 3 d.f.
probability =     0.0034
```

EN LA ULTIMA SEMANA COMPROBAR SI LA ULTIMA SEASON ES LA QUE DIFIERE DE LAS OTRAS

```
. . ranksum long_term_fac if week ==2  & (season ==1| season ==4)  ,
by(season)

Two-sample Wilcoxon rank-sum (Mann-Whitney) test
```

```
      season |      obs    rank sum    expected
-------------+---------------------------------
   2016-2017 |        6        54.5          39
   2019-2020 |        6        23.5          39
-------------+---------------------------------
    combined |       12          78          78

unadjusted variance       39.00
adjustment for ties       -0.68
                     ----------
adjusted variance         38.32

Ho: long_t~c(season==2016-2017) = long_t~c(season==2019-2020)
             z =   2.504
    Prob > |z| =   0.0123

. . ranksum long_term_fac if week ==2  &   (season ==2| season ==4)  ,
by(season)

Two-sample Wilcoxon rank-sum (Mann-Whitney) test

      season |      obs    rank sum    expected
-------------+---------------------------------
   2017-2018 |        6        53.5          39
   2019-2020 |        6        24.5          39
-------------+---------------------------------
    combined |       12          78          78

unadjusted variance       39.00
adjustment for ties       -0.68
                     ----------
adjusted variance         38.32

Ho: long_t~c(season==2017-2018) = long_t~c(season==2019-2020)
             z =   2.342
    Prob > |z| =   0.0192

. . ranksum long_term_fac if week ==2  &   (season ==3| season ==4)  ,
by(season)

Two-sample Wilcoxon rank-sum (Mann-Whitney) test

      season |      obs    rank sum    expected
-------------+---------------------------------
   2018-2019 |        6          57          39
   2019-2020 |        6          21          39
-------------+---------------------------------
    combined |       12          78          78

unadjusted variance       39.00
adjustment for ties       -0.55
                     ----------
adjusted variance         38.45

Ho: long_t~c(season==2018-2019) = long_t~c(season==2019-2020)
             z =   2.903
    Prob > |z| =   0.0037
```

EN LA ULTIMA SEASON COMPARAR LAS TRES SEMANAS

Kruskal-Wallis equality-of-populations rank test

week	Obs	Rank Sum
41-6	18	287.00
7-12	6	131.00
13-18	6	47.00

```
chi-squared =     7.702 with 2 d.f.
probability =     0.0213

chi-squared with ties =     7.776 with 2 d.f.
probability =     0.0205
```

OTHER

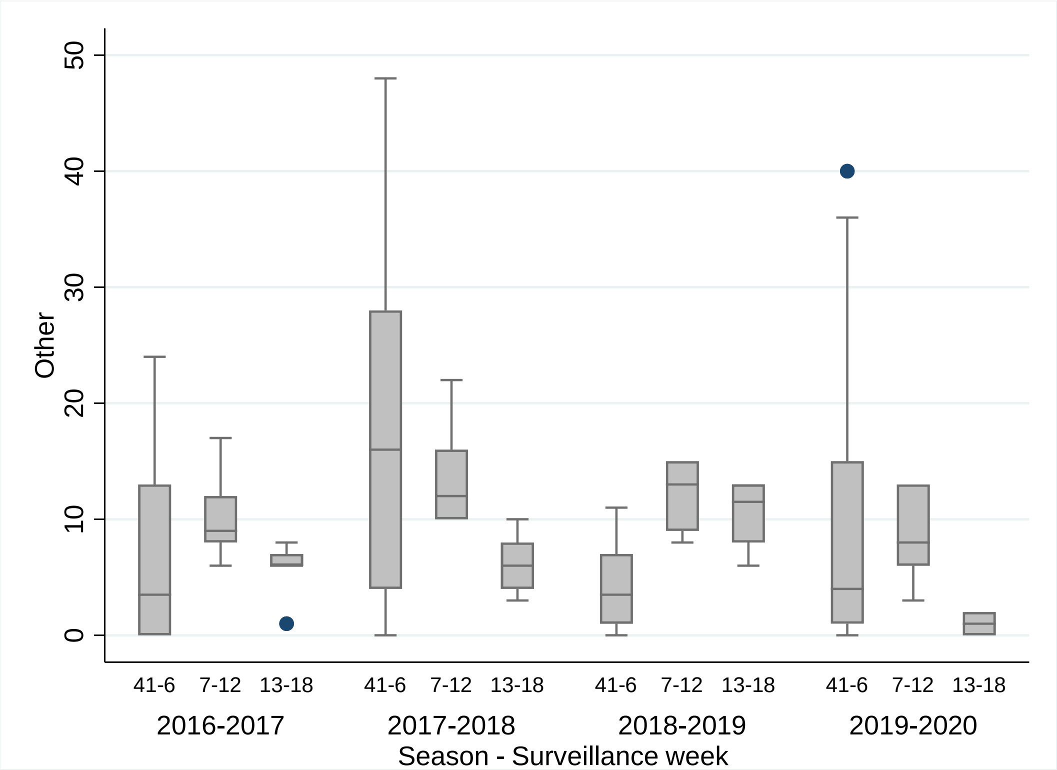

COMPARACIÓN DE LAS ESTACIONES SEGÚN SEMANA

```
. . kwallis other if week ==0, by(season)
```

Kruskal-Wallis equality-of-populations rank test

season	Obs	Rank Sum
2016-2017	18	582.50
2017-2018	19	922.50
2018-2019	18	537.50
2019-2020	18	658.50

```
chi-squared =     8.538 with 3 d.f.
probability =     0.0361

chi-squared with ties =     8.650 with 3 d.f.
probability =     0.0343

. . kwallis other if week ==1, by(season)

Kruskal-Wallis equality-of-populations rank test
```

season	Obs	Rank Sum
2016-2017	6	62.00
2017-2018	5	81.00
2018-2019	6	85.50
2019-2020	6	47.50

```
chi-squared =     5.115 with 3 d.f.
probability =     0.1636

chi-squared with ties =     5.171 with 3 d.f.
probability =     0.1597

. . kwallis other if week ==2, by(season)

Kruskal-Wallis equality-of-populations rank test
```

season	Obs	Rank Sum
2016-2017	6	74.00
2017-2018	6	82.00
2018-2019	6	120.00
2019-2020	6	24.00

```
chi-squared =    15.587 with 3 d.f.
probability =     0.0014

chi-squared with ties =    15.793 with 3 d.f.
probability =     0.0013
```

EN LA ULTIMA SEMANA COMPROBAR SI LA ULTIMA SEASON ES LA QUE DIFIERE DE LAS OTRAS

```
. . ranksum other if week ==2  & (season ==1| season ==4)  , by(season)
```

```
Two-sample Wilcoxon rank-sum (Mann-Whitney) test

      season |      obs    rank sum    expected
-------------+---------------------------------
   2016-2017 |        6          54          39
   2019-2020 |        6          24          39
-------------+---------------------------------
    combined |       12          78          78

unadjusted variance       39.00
adjustment for ties       -1.64
                     ----------
adjusted variance         37.36

Ho: other(season==2016-2017) = other(season==2019-2020)
             z =   2.454
    Prob > |z| =   0.0141

.
.
. . ranksum other if week ==2  &   (season ==2| season ==4)  , by(season)

Two-sample Wilcoxon rank-sum (Mann-Whitney) test

      season |      obs    rank sum    expected
-------------+---------------------------------
   2017-2018 |        6          57          39
   2019-2020 |        6          21          39
-------------+---------------------------------
    combined |       12          78          78

unadjusted variance       39.00
adjustment for ties       -1.36
                     ----------
adjusted variance         37.64

Ho: other(season==2017-2018) = other(season==2019-2020)
             z =   2.934
    Prob > |z| =   0.0033

. . ranksum other if week ==2  &   (season ==3| season ==4)  , by(season)

Two-sample Wilcoxon rank-sum (Mann-Whitney) test

      season |      obs    rank sum    expected
-------------+---------------------------------
   2018-2019 |        6          57          39
   2019-2020 |        6          21          39
-------------+---------------------------------
    combined |       12          78          78

unadjusted variance       39.00
```

```
adjustment for ties        -1.23
                      ----------
adjusted variance          37.77

Ho: other(season==2018-2019) = other(season==2019-2020)
             z =   2.929
    Prob > |z| =   0.0034
```

EN LA ULTIMA SEASON COMPARAR LAS TRES SEMANAS

```
Kruskal-Wallis equality-of-populations rank test

  +-------------------------+
  |  week | Obs | Rank Sum |
  |-------+-----+----------|
  |  41-6 |  18 |   295.50 |
  |  7-12 |   6 |   123.00 |
  | 13-18 |   6 |    46.50 |
  +-------------------------+

chi-squared =     6.781 with 2 d.f.
probability =     0.0337

chi-squared with ties =     6.862 with 2 d.f.
probability =     0.0324
```

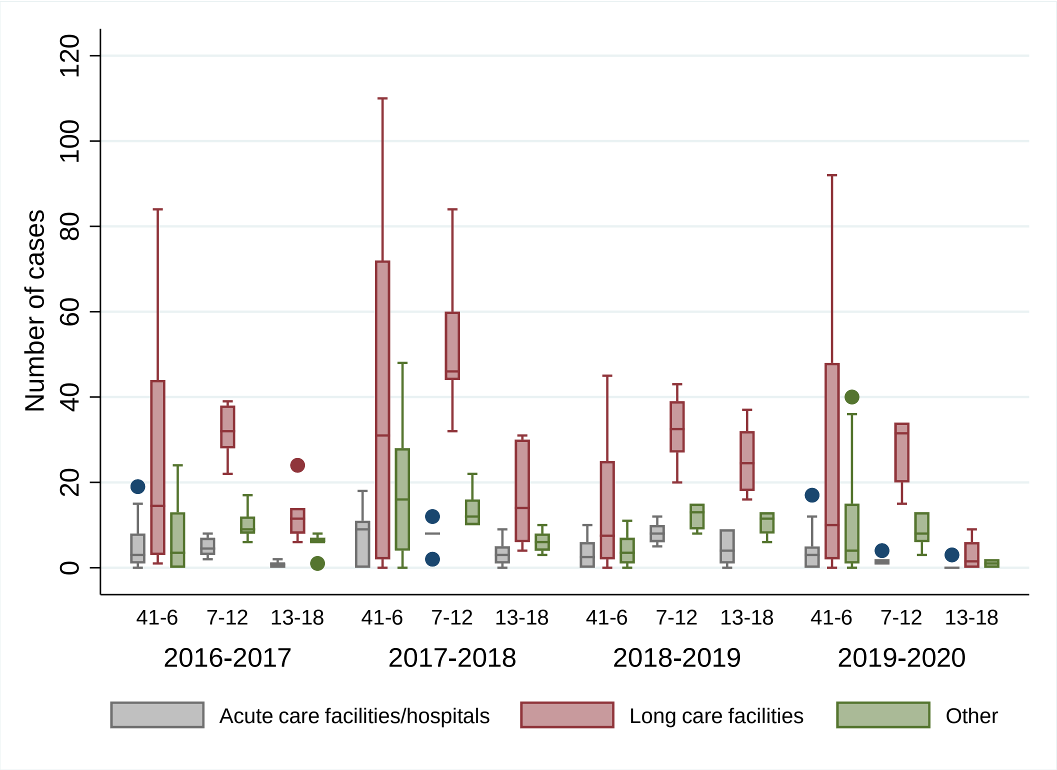

```
. . by week, sort : tabstat A_uns A_H3N2 A_H1N1 B hospitals long_term_fac other , statisti
> cs( median p25 p75 ) by( Season )
```

-> week = 41-6

Summary statistics: p50, p25, p75
by categories of: Season (season)

Season	A_uns	A_H3N2	A_H1N1	B	hospit~s	long_t~c	other
2016-2017	226.5	440.5	4	14.5	3	14.5	3.5
	31	140	4	7	1	3	0
	1222	1225	4	39	8	44	13
2017-2018	523	506	33	473	9	31	16
	71	153	7	47	0	2	4
	1675	808	54	1802	11	72	28
2018-2019	1042.5	35	519.5	15.5	2.5	7.5	3.5
	244	18	295	8	0	2	1
	1613	93	828	40	6	25	7
2019-2020	372	77	57.5	495	3	10	4
	57	43	20	39	0	2	1
	1620	139	370	1463	5	48	15
Total	523	114	33	40	3	12	5
	71	50	4	14	0	2	1
	1516	467	315	473	8	45	15

-> week = 7-12

Summary statistics: p50, p25, p75
by categories of: Season (season)

Season	A_uns	A_H3N2	A_H1N1	B	hospit~s	long_t~c	other
2016-2017	889.5	812.5	12	181	4.5	32	9
	629	347	7	128	3	28	8
	1291	935	14	242	7	38	12
2017-2018	1023	265	73	1714	8	46	12
	807	246	51	1387	8	44	10
	1437	279	76	2057	8	60	16
2018-2019	1075.5	310	214.5	67.5	8	32.5	13
	990	236	197	40	6	27	9
	1116	357	244	90	10	39	15
2019-2020	1754.5	72	312	1219.5	1	31.5	8
	1552	58	255	1015	1	20	6
	1772	76	354	1559	2	34	13
Total	1085	265	154	302	5	32	10
	832	81	17	97	2	28	8
	1552	357	255	1387	8	39	14

-> week = 13-18

Summary statistics: p50, p25, p75
by categories of: Season (season)

Season	A_uns	A_H3N2	A_H1N1	B	hospit~s	long_t~c	other
2016-2017	191.5	66	4	318.5	1	11.5	6
	113	47	0	309	0	8	6
	273	134	6	347	1	14	7
2017-2018	336	105.5	37	411	3	14	6
	215	62	29	238	1	6	4
	436	143	49	661	5	30	8
2018-2019	685	284.5	48.5	168	4	24.5	11.5
	448	244	41	157	1	18	8
	939	387	121	175	9	32	13
2019-2020	9.5	1	5	16.5	0	1.5	1
	4	0	0	11	0	0	0
	48	5	11	97	0	6	2
Total	244.5	78.5	26	216	1	11.5	6
	89	28.5	4	139.5	0	6	2
	442	202	41.5	321.5	3	22.5	8

.
